# Impact of Diet and Nutrition in Patients with Acne Vulgaris

**DOI:** 10.3390/nu16101476

**Published:** 2024-05-14

**Authors:** Izabella Ryguła, Wojciech Pikiewicz, Konrad Kaminiów

**Affiliations:** 1 Faculty of Medical Sciences in Katowice, Medical University of Silesia, 40-752 Katowice, Poland; izabella.ryg@gmail.com; 2 Collegium Medicum—Faculty of Medicine, WSB University, 41-300 Dabrowa Gornicza, Poland; wojciech.pikiewicz@wsb.edu.pl

**Keywords:** acne, diet, nutrition, skin, dermatology disease

## Abstract

Acne vulgaris is a widespread a chronic inflammatory dermatosis that affects millions of people around the world, which has a significant influence on patients’ standard of living. The progression of this dermatosis results in the appearance of inflammatory and non-inflammatory changes, and, in severe cases, disfiguring scars and hyperpigmentation. The aetiopathogenesis of acne is complex. It involves a complex interaction of many different factors, both endo- and exogenous in their effect on the hair and sebaceous unit. Genetic predisposition, hormones, the skin and gut microbiome, psychological stress, air pollutants, aggressive facial products, and certain medications are cited as factors influencing acne formation. The link between nutrition and acne is extensively debated for many years and is still relatively controversial. Diet is commonly recognised to have a direct relationship with certain biochemical markers and the transcription of genes related to sebaceous gland function, and the proliferation of bacteria and inflammation that encourage the progression of the disease. In this review, the authors take a closer look at the existing scientific reports on the involvement of nutrition in the development of acne vulgaris.

## 1. Introduction

Acne vulgaris has been described as a chronic and widespread inflammatory skin disease that is regarded as the most common skin condition in adolescence and early adulthood. It affects 80–100% of people aged from 11 to 30 years, 85% of whom have a mild course, while 15% of sufferers develop acne of a particularly severe severity [1,2,3]. Moreover, in the general population, 9% of the worldwide population struggles with acne [4]. The aetiology of acne appears likely to be complex and includes genetic, hormonal, and environmental factors, among others [5]. This dermatosis is manifested by flammable changes like papules, abscesses, or pustules, typically localised on the face, arms, and thorax, and non-inflammatory lesions like open or closed comedones [6,7]. According to the morphological characteristics and severity of the lesions, the various clinical forms of acne are identified in the following: comedonal acne, with predominantly closed and/or open comedones; papulopustular acne, characterised by the existence of non-inflammatory lesions (papules and/or pustules); acne with intense inflammation (counting nodular acne and clustered acne) with the presence of nodules or cysts; scarring acne, characterised by the presence of scars; fulminant acne (acne fulminans); and purulent acne (acne excoriée). [8]. Microscopic lesions called microcalcifications are believed to form the basis of non-inflammatory and inflammatory acne lesions. Their formation occurs through hormonal disturbances. Keratinocyte hyperproliferation, the growth of sebaceous groups, and the excessive production of sebum of abnormal composition are observed. The increased keratinisation of the hair and sebaceous unit mouths results in sebum retention and the formation of a hyperkeratotic plug in the hair follicle funnel. Sebaceous sebum creates a favourable environment for the development of Cutibacterium acnes (formerly known as Propionibacterium acnes), implying inflammation and the transformation of microcalcifications into acne lesions [1,9,10,11,12,13]. In summary, the pathogenesis of acne is ascribed to four crucial determinants: (1) excessive sebum secretion; (2) Cutibacterium acnes hyperproliferation; (3) the excessive keratinisation of hair and sebaceous follicles; and (4) inflammatory mechanisms [14]. In the course of acne, especially severe acne, skin lesions in the form of scars or hyperpigmentation may occur, leading to the significant disfigurement of the body, especially the skin of the face of patients [15]. The disease leads to a significant reduction in the sufferer’s well-being, resulting in reduced self-regard and self-confidence, trouble with public interactions, and psychological distress [16]. The strongly negative impact that this dermatosis has on patients’ quality of life and its multifactorial nature have led researchers to search for innovative and effective therapeutic approaches, resulting in decreasing the exacerbation of cutaneous changes and improved patient well-being [17]. In the last few years, the focus on the influence of certain dietary components on the development of acne and their possible elimination to reduce acne lesions has increased. The purpose of this analysis is to summarise the existing scientific knowledge concerning the influence of diet on the pathogenesis of acne vulgaris.

A search was conducted in the PubMed, Medline, and Google Scholar databases to identify the literature related to the impact of diet and nutrition in the pathogenesis of acne vulgaris. The following terms were used in the searching process: “acne”, “diet”, “nutrition”, ‘’food factors’’, ‘’milk”, ‘’chocolate’’, and ‘’fatty acids’’. Manuscripts were reviewed for titles, abstracts, and the entire text based on the following criteria: (1) original papers; (2) reviews; and (3) impact of diet and/or nutrition on the pathogenesis of acne vulgaris as a key topic of the paper. Authors excluded methodological studies, editorials, commentaries, and letters. The analysis was conducted in the following steps. The first step was related to the analysis of selected papers based on titles and abstracts, the second step was connected with the analysis of full-text papers, and the last step included the analysis of the collected data.

## 2. Food Factors in the Pathogenesis of Acne Vulgaris

Dietary management is a part of the large amount of attention being paid to the aetiology, therapy, and prevention of dermatological illnesses, including acne vulgaris. The research has demonstrated that the prevalence of acne is significantly greater in Western populations than in non-Western populations, which is considered to be due to differences in diet. Western diets often lack long-chain omega-3 fatty acids, but include large amounts of refined carbohydrates. An imbalance between omega-6 and omega-3 fatty acids is considered to be strongly involved in the development of acne [18]. This diet is rich in dairy products, refined carbohydrates, chocolate, and saturated fats, which can exacerbate acne by activating aliment-derived metabolic cues [19]. Saturated fatty acids, which are abundant in the Western diet, induce inflammation through TLR2/IL-1B receptor expression promoting Th17 lymphocyte diversity. This enhances IL-17A secretion, which promotes the hyperproliferation of keratinocytes and decreased diversification [20,21].

Furthermore, the Western diet, characterised by a higher consumption of dairy products and a high glycaemic index (GI), influences the levels of hormones involved in the aetiology of acne. The GI is a scoring system on a scale of 1 to 100 to determine how quickly carbohydrates from a given product are digested, absorbed, and metabolised. Diets with a relatively high GI (being above 55) have been shown to be associated with mediocre glycaemic control, increased postprandial insulin levels, and elevated IGF-1 levels, whereas diets with a relatively low GI result in decreased fasting IGF-1 [22,23,24]. Equally, people who regularly consume dairy have elevated serum IGF-1 and insulin levels in comparison to those who do not consume dairy, and the consumption of whey or casein, the protein components of dairy, is linked to increased IGF-1 and insulin values [25,26,27].

Excessive sebum production results from the increased activity of androgen hormones, insulin-like growth factor-1 (IGF-1), and insulin [28]. Hyperinsulinaemia significantly increases circulating levels of IGF-1. IGF-1 is known to be a mythogen synthesised mostly in the liver and promotes sebaceous cell proliferation and lipogenesis in the sebaceous glands. IGF-1 also increases androgen levels and suppresses the production of the IGF-binding proteins IGFBP-1, IGFBP-3, and sex hormone-binding globulin (SHBG). The inhibition of the production of these proteins implies the increased availability of androgens and IGF-1, thus enhancing the exacerbation of acne [7,29,30,31].

Insulin and IGF-1 were demonstrated to reduce the levels of nuclear forkhead box protein O1 (FOXO1), resulting in its translocation from the cell nucleus to the cytoplasm. Correctly, the inhibition of protein synthesis, cell growth, and lipid metabolism is cited as the role of FOXO1 being in the cell nucleus. FOXO1 increases the expression of sestrin 3, resulting in the inhibition of mTORC1, while the translocation of FOXO1 into the cytoplasm results in the activation of the mammalian target of rapamycin complex 1 (mTORC1). Sestrin 3 assists in regulating blood glucose levels and supports insulin sensitivity, ehile mTORC-1 is a serine/threonine kinase which functions as a controller of height, proliferation, lipid synthase, and transcription of proteins [32,33]. The function of mTORC1 on the development of acne lesions is to mediate increased sebaceous gland proliferation, lipid synthesis, and keratinocyte proliferation, both alone and through androgen-mediated processes [34,35]. An increase in mTORC-1 activation also involves the transcription of the sterol regulatory element binding protein (SREBP) gene. It is responsible for regulating the production of fatty acids, cholesterol, phospholipids, and triglycerides. It also promotes sebaceous gland enlargement and lipogenesis [29,36]. Then, Cutibacterium acnes, via triacylglycerol lipase, transforms the triacylglycerols available in normal sebum to free fatty acids: palmitic acid, oleic acid, and sapenoic acid. This leads to the development of biofilm production [37]. This efficient inhibition of FOXO1 additionally enhances the action of androgens, both by enabling the uncontrolled activation of the mTORC1 route and by enhancing the displacement of the androgen receptor to the nucleus, in which it has its effect [32]. It is noteworthy that an increase in S6 kinase-1 secretion is observed in response to the reduced mTORC-1 activity and reduced FOXO1 levels. S6 kinase-1-mediated phosphorylation of insulin receptor substrate-1 implies a decrease in insulin/IGF-1 signalling and, ultimately, causes insulin resistance [30,38]. Moreover, aminoalkanoic acid widely distributed in meat and dairy proteins, leucine, also has the ability to activate mTORC1 [34].

It is worth noting that a large body of evidence also indicates that an abnormal diet has a fundamental effect on dysbiosis in the gut flora, being a cause of metabolic and inflammatory skin diseases, including severe inflammatory forms of acne [39,40,41]. Abnormalities in the gut microbiome contribute to the pathogenesis of acne, mainly via a modified mTOR route and enhanced gut barrier permeability [42,43].

**Figure 1:**
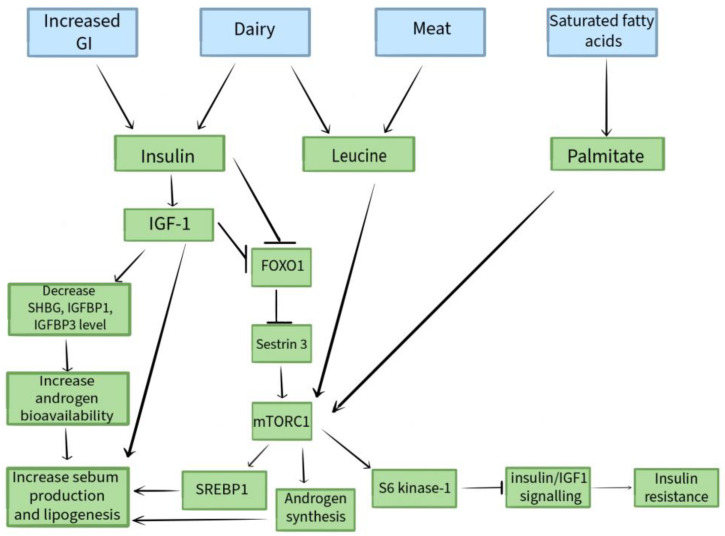
Schematic presentation of the influence of diet on the pathogenesis of acne vulgaris.

Figure 1 shows a schematic presentation of the influence of diet on the pathogenesis of acne vulgaris. 

## 3. Nutrients with a Potentially Negative Impact on Acne

### 3.1. Milk and Dairy Products

Milk is a supremely specialist functional nutrition designed through evolution to support anabolic reactions and newborn mammalian growth [19,44]. Milk consumption significantly increases the amount of IGF-1 [14]. We know that 80% of the milk protein from cows is casein, while 20% is whey protein. Whey proteins have been involved in the insulinotropic action of milk, and casein is more likely to stimulate IGF-1 than whey [45]. Whey proteins contain six growth factors: platelet-derived growth factor (PDGF), tumour growth factor (TGF), fibroblast growth factor-1 (FGF-1), FGF-2, IGF-1, and IGF-2, which stimulate insulin secretion in the pancreas. Athletes use whey proteins for oral supplementation in order to enhance muscular weight [30]. The hyperinsulinaemia induced by the insulinotropic function of such proteins also raises IGF-1 concentrations, providing a possible reason to explain why people consuming these supplements struggle with the appearance or exacerbation of acne lesions [46,47]. Milk acts as an ideal fuel for FOXO1/mTORC1/SREBP-1c-regulated sebaceous gland hypertrophy and sebaceous lipogenesis. The enhanced generation of IGF-1 as a result of protein consumption coincides with excessive IGF-1 signalling during puberty, which explains the sooner initiation of puberty and the maintenance of acne in the third decade of age among people ingesting that drink [19].

Furthermore, cow’s milk, mainly from pregnant cows, contains not only progesterone from the placenta, but other precursors of dihydrotestosterone, such as 5α-pregnandione and 5α-androstendione. These chemicals are easily converted by enzymes to DHT, as are the enzymes needed for this transformation that are available in the hair and sebaceous unit. It is notable that dihydrotestosterone is the most biologically reactive kind of testosterone, which is important in the formation of acne lesions via growing the expression of androgen receptors and, consequently, activating mTORC-1 [30,48,49,50].

It should be noted that leucine, which supports mTORC1 activation, is most abundant not in animal protein sources (8%), but in whey protein (14%) [51]. 

There are numerous articles in the bibliography that address the effect of milk and dairy products on the pathogenesis of acne lesions [46,52,53,54,55,56,57,58,59,60,61,62,63,64,65,66,67,68,69,70]. The authors collected and divided the studies according to the age of the research group. Studies involving adolescents, young adults (understood as those aged 18–25), and adults were distinguished. A group of research reports in which the age range of the study participants was relatively wider were also identified.

Ulvestad M. et al. conducted a questionnaire-based study on the baseline dairy intake and the presence of acne after 3 years. The study was conducted among 2489 Norwegian adolescents aged 15–16 years. The consumption of at least two glasses of full-fat-milk-based product daily was related with the presence of medium to extreme acne [55]. 

A group of researchers, Adebamowo C.A. et al., conducted a retrospective cohort study in which 47,355 female attendees replied to and fulfilled a questionnaire on nutriment abundance in high school. This analysis includes women who fulfilled the questionnaire and responded to a question about doctor-diagnosed extreme teenage acne in 1989. A positive corelation with acne was found for the consumption of whole and skimmed milk. Cottage cheese and cream cheese were also positively connected with acne [58]. From this retrospective study, attempts to conduct two further studies, on a female adolescent population and a male adolescent population, were born.

Adebamowo C.A. et al. set out to determine the relation between the dietary consumption of dairy products and acne through 6094 adolescent girls. This study showed that whole milk, low-fat milk, skimmed milk, or chocolate milk was connected with acne [59].

Adebamowo C.A. et al. also set out to determine the relation between the dietary consumption of dairy products and acne among adolescent males. In this prospective cohort research, 4273 boys of baseline age between 9 and 15 years were surveyed using a questionnaire. A positive association between the consumption of skimmed milk and the incidence of acne was proven. Whole milk, 2% milk, and low-fat milk were not associated with acne [60].

Okoro E. et al. performed a physical examination and data analysis on 464 Nigerian adolescents. Of these, 299 were found to have acne vulgaris, and its prevalence was higher among those who consumed milk as a beverage at least once a day [61].

LaRosa et al. managed a case-control study through 225 attendees aged 14 to 19 years, with or without moderate acne. Acne lesions were assessed during a dermatological examination and the subjects’ diets were evaluated through the Nutrition Data System for Research software. The results indicated that intake of low-fat/defat milk, but not whole milk, was positively related with acne incidence [63].

It is worth noting that AlKhabbaz M. et al. performed a cross-sectional analysis among 714 adolescents, in which they found that triggers that were not strongly related to the development of acne vulgaris included semi-skimmed milk, full-fat cream cheese, low-fat cheese, white cheese, yoghurt, ice cream, and other dairy products [70].

Pereira Duquia R. et al. conducted a study involving 2201 18-year-old men post-military in southern Brazil to investigate the prevalence of acne and factors associated with it. Researchers showed in this study that, although the crude analysis showed a relation between daily whole milk consumption and the incidence of inflammatory acne, this association was only found in the proximate investigation. Even though the outcomes of the corrected studies were not statistically relevant, the prevalence rates of daily whole milk intake may indicate a poor positive correlation with the incidence of inflammatory acne [64].

Burris J. et al. conducted a study that enrolled 248 participants aged 18 to 25 years (115 men and 133 women). Compared to participants without acne or with soft acne, attendees with medium to extreme acne reported a higher intake of the amount of servings of milk per day, with the implication that milk may trigger or exacerbate the development of acne [65].

Cegniz F. et al. described the case of six males in good health with an average age of 18 years who had acne found exclusively on the torso subsequent to consuming whey protein supplements for faster bodybuilding [66].

In a descriptive observational study conducted by Pontes Tde, C. et al. in gyms and a dermatological department, 30 patients aged 18–30 years were studied to estimate the association between the intaking of protein supplementation and the occurrence or deterioration of acne vulgaris. Every attendee was tested for acne on three occasions (before starting supplementation, and then one month and two months after starting supplementation). This study showed that the use of protein and calorie supplementation over a two-month period was associated with acne. This impact was more pronounced among females and among people with no present acne changes and without a family history of acne [46].

Semedo D. et al. designed a cross-sectional analysis involving 1055 adults aged 20 to 60 years living in Portugal (Porto). Among the subjects, 61.5% had acne. Only 36.8% of adults who went through acne were conscious of their disease. The outcome of the research confirmed that acne appears to be associated with a higher consumption of full-fat and reduced-fat milk. At this point, it is also worth noting the strong influence that smoking has on the pathogenesis of acne. As many as 62.3% of the study participants who smoked tobacco suffered from acne lesions [56].

Penso et al. designed a prevalence analysis involving 24,452 French adult participants. Milk intake was shown to be connected with adult acne incidence [53].

However, it needs to be added that Heng A.H.S et al. studied 3888 patients (2090 cases with acne and 1798 controls) through a questionnaire and by assessing acne lesions on a physical examination. The patients ranged in age from 17 to 71 years. Their study led to the interesting conclusion that frequent milk consumption was associated with a preventive outcome for medium acne, whereas common butter intake had a harmful impact on the degree of acne scarring [68].

Juhl C.R. et al. examined the thesis that genetically predisposed milk intake is related to adult acne with a Mendel randomisation scheme.

LCT-13910 C/T is related with lactase perseverance in Northern Europeans. The association between milk consumption, LCT-13910 C/T, and acne was investigated in 20,416 mature persons from the Danish General Population Survey (GESUS). The study showed that no observational or genetic connection was found between milk consumption and acne [69].

Aalemi et al. managed a case–control study to determine the relationship of dairy intake with acne among Kabul residents. The research contained 279 people with acne and 279 control subjects. The age of the subjects was between 10 and 24 years old. This study showed that the consumption of whole milk at least three days per week was related with moderate to severe acne, and that the relation for skimmed milk was not so greatly expressed as that for regular milk [52].

Juhl C. R. et al., in their meta-analysis, decide to assess the relationship between dairy consumption and acne among children, adolescents, and young adults in a data-based analysis. The review included 23,046 patients with acne and 55,483 representing a control group aged 7 to 30 years. The conclusion of this study was that any dairy such as milk, yoghurt, and cheese was related with an enlarged odds ratio (OR) for acne in those aged 7 to 30 years [54]. The paper presented by Juhl et al. is a meta-analysis, so the authors conclude that it is worth interpreting these results with caution due to the heterogeneity and bias of the studies.

In a case–control study led by a group of researchers headed by Di Landro A. et al., 205 acne sufferers and 358 control subjects (people without acne or with mild acne, presenting for a dermatological consultation for a reason other than acne) were analysed via questionnaires and physical examination. The study participants were aged between 10 and 24 years. The conclusion of this study was that the risk of medium to extreme acne increased with increased milk intake in those drinking more than three portions per 7 days. This relationship was more pronounced for skimmed milk than for full-fat milk [57].

In a case–control study created by Kara Y. A. et al., including 53 persons who suffered from acne vulgaris and 53 controls, acne was assessed by a dermatologic specialist based on Global Acne Grading System calculations. The intake of various dietary components, including milk and milk products, was also calculated. The results suggest that there is a statistically positive correlation between acne severity and cheese consumption. It is known that, during milk processing, extra testosterone is produced from precursors such as androstendione and estrone, depending on the fermentation stage in milk products like cheese [62]. 

Interestingly, the study by Say Y.H. et al. involved 1840 patients, of whom 1117 were acne sufferers and 723 were controls. Patients completed questionnaires assessing, among other things, their eating habits. Their acne lesions were assessed by trained staff for severity and degree of scarring. The study found that the frequent intaking (on most or all days) of nutriments typically eaten for breakfast, such as butter, milk, cereal, and probiotic drinks, reduced the risk of acne and larger acne scars [67].

In Table 1, the authors summarised selected studies on the effect of milk and dairy products on the pathogenesis of acne vulgaris.

It is worth noting that there are conflicting data on which type of milk is involved in the pathogenesis of acne. In a study by Ulvestad M. et al., a high intake of full-fat dairy products was associated with acne, whereas an association between acne lesions and the consumption of skimmed or semi-skimmed dairy products was not found [55]. The opposite trend can be seen in scientific reports created by Juhl C.R et al. [54], Di Landro A et al. [57], LaRosa et al. [63], and Adebamowo C.A. et al. [58]., in which the consumption of low-fat/defat milk correlated more strongly with the appearance of acne lesions than full-fat milk. What might this conflicting information be due to? It is generally suggested that the acne-causing effects of dairy are unlikely to be due to the fat content, but rather to the hormones and other bioactive molecules contained in milk. Skimmed milk contains hormonal components or endogenous hormone-influencing agents in sufficient quantities to produce biological effects in consumers. It is worth pointing out that it is not only saturated fats that are included in milk fat. This fat also contains substances that have a beneficial effect on metabolism and the reduction of insulin resistance. These substances are medium-chain fatty acids. Moreover, bioactive branched-chain fatty acids, conjugated linoleic acid and monounsaturated fatty acids, oleic acid, and transpalmitoleic acid are included in milk fat, exerting a positive effect on the metabolic profile [54,55,57,58,63].

### 3.2. Chocolate

Research on the effects of chocolate on skin conditions in acne are often contentious and inaccurate because of the presence of additional, non-cocoa ingredients (milk, sugar, etc.) in chocolate products [71]. Dark chocolate is known to be abundant in phenolic antioxidants, principally flavonoids, which were repeatedly demonstrated to have a preventive effect in the prevention of cardiovascular disease and diabetes, but a growing number of studies suggest that chocolate intake is associated with the progression of acne vulgaris among adolescents [72,73,74,75,76,77]. Theses were proposed to clarify the potential impact of chocolate intake on acne symptoms. A suggestion is that the saccharides present in chocolate (and milk) induce insulin excretion, triggering signalling routes that, ultimately, implicate enhanced keratinocyte proclivities, resulting in acne lesions [2]. In addition, cocoa ingredients were demonstrated to boost the secretion of inflammatory cytokines, including IL1β. IL1β expression has been shown to precede keratinocyte keratinisation, which is directly linked to the eventual exfoliation of corneocytes, the severity of which can be interpreted as a pro-inflammatory phenomenon [78,79,80].

Chalyk et al. evaluated the effect of the continuous consumption of semi-sweet chocolate on the structural attribute of residual skin surface components (RSSCs) taken from the face of young, mid-aged men. For four weeks, 17 young-aged men (20 to 30 years) and 16 middle-aged men (45 to 75 years) ingested 10 g of dark chocolate (containing 70% cocoa) daily. Samples taken before the start of the study and after four weeks of chocolate consumption were assessed. The results were as follows: chocolate consumption increased corneocyte exfoliation in both age groups, but the rise appeared to be statistically relevant only among young men. Furthermore, chocolate consumption for four weeks resulted in a substantial growth in Gram-positive micro-organisms on the facial skin surface in both young and middle-aged men [72].

Caperton et al. managed a double-blind, placebo-controlled work of research to evaluate the effect of chocolate on acne intensifying in men aged 18 to 35 years that have a medical history of acne. The investigation included 14 participants. The men were allocated to ingest capsules loaded with non-sweetened 100% cocoa, hydrolysed gelatine dust, or a mix of both. Changes were assessed and photographs were taken at the start of the study, on day four, and on day seven of the research. A statistically considerable growth in the average figure of all acne-like lesions (blackheads, papules, pustules, and nodules) was found on a comparison with the initial results on both days 4 and 7. There was a positive Pearson connection coefficient of low strength between the quantity of chocolate consumed by every participant and the amount of skin changes developed in every patient between baseline and day 4, whilst there was a positive correlation of medium strength between baseline and day 7. These results would indicate that chocolate consumption positively correlates with the severity of acne lesions among men prone to this dermatosis [81].

Fifty-seven patients struggling with acne vulgaris and fifty-seven controls were enrolled in a retrospective work of research managed by Suppiach et al. The attendees were generally aged fourteen or over. The severity of acne lesions was judged by a specialist of dermatology and the subjects’ dietary habits were surveyed. The analysis showed that chocolate consumption was significantly greater among the sufferers than in the controls [82].

Moreover, 3826 sick people with acne vulgaris and 759 controls were enrolled in the research conducted by Karadağ et al. Acne lesions were assessed by a dermatologist, and, in addition, the subjects completed questionnaires on frequency and dietary habits. Based on these surveys, a positive relation was noticed between the intake of various products, including chocolate, and the intensity of acne lesions [83].

Vongraviopap et al. enrolled 25 acne-prone men in their study. Participants consumed 25 g of dark chocolate (99% cocoa) every day for four weeks. The assessment of acne lesions took place weekly. This study concluded that dark chocolate taken in normal quantities for 4 weeks can worsen acne among men prone to this dermatosis [84].

In Table 2, the authors summarised selected studies on the effect of chocolate on the pathogenesis of acne vulgaris.

### 3.3. Saturated Fatty Acids and Trans Fatty Acids

A high content of fats of animal origin in the diet may have the potential to become a nutritional trigger of acne. Saturated fatty acids, which include, for example, Palmitoleic, Stearylic, or Myristic acid, and trans fatty acid isomers, the primary source being hydrogenated plant fats contained in margarine, confectionery, or fast food (for example, chips and pizza), negatively affect the skin affected by acne vulgaris [85,86]. The high content of palmitic acid leads to the progression of dermal infection, increased blackheads, and increased sebum production. This is due to the impact on the generation of pro-inflammatory cytokines: IL-1β and IL-1α. It is also known that palmitate, a main saturated fatty acid, activates the mTORC1 pathway and reinforces its lysosomal displacement, while the eicosapentaenoic acid (EPA) present in fish, which belongs to the omega-3 family of acids, inhibits mTORC1 activation. Saturated trans fats through the stimulation of pro-inflammatory TLR2/TLR4 signalling via the receptor for TNF-related factor 6 may contribute to the inflammatory response of hair and sebaceous units mediated by dietary nutrients [19,87].

In the aforementioned cross-sectional study that Penso et al. conducted as an element of the NutriNet-Santé study, 24,452 French residents participated. The attendees answered an online survey about the condition of their acne lesions and diet. Associations between dietary behaviour and present or previous acne were examined in polynomial logistic regression models corrected for possible disturbing factors (for example, age). The research showed a relevant relationship between present acne and the intake of fatty foods [53].

Aalemi et al. conducted a case–control study to determine the relation of dairy intake with acne among Kabul residents. The research contained 279 sick people struggling with acne and 279 controls. This study showed that the consumption of products such as crisps, pizza, or red meat showed a positively related relationship with the incidence of acne changes [52].

A study by Wei et al. enrolled 5696 students (2920 patients and 2776 controls) who completed relevant questionnaires. On this basis, acne risk factors were identified, among which fried food was listed [88].

Abo El-Fetoh et al. set out to describe risk factors for acne vulgaris among 400 male students with an average age of 15 years old. Data for the research were collected by personal interview and questionnaire completion. Risk factors for acne included the consumption of fatty meals [89].

In Table 3, the authors summarised selected studies on the effect of fatty foods on the pathogenesis of acne vulgaris.

### 3.4. Other Products That Can Worsen Acne

There are also reports suggesting the impact of other foods on the evolution of acne vulgaris. 

Alcohol intake seems to play a role as a possible determinant for acne. Many cross-border investigations in European and Asian populations have demonstrated that alcohol abusers have a markedly greater prospect of acne than abstainers [67,68,90,91,92]. However, Shen et al. showed that the prevalence of acne among people over 25 years of age was 5.5% in chronic alcoholics, 5.8% in moderate drinkers, and 5.5% in abstinents, which would suggest that alcohol does not seem to be a determinant for acne among adults [93]. It is worth noting alcohol has been demonstrated to enhance both testosterone values and the secretion of the proinflammatory cytokines. Moreover. in the long term, it damages the immune system, linking bacteria proclivity to changes in the dermal microbiome and acne aggravation, and. when expelled with perspiration, functions as a food component for C. acnes [30,92].

There is also scientific evidence suggesting that salt and salty snacks influence the formation of acne changes [94,95,96,97]. A study by Darouti et al. showed that 76% of acne sufferers consumed more NaCl than recommended by the joint ‘FAO/WHO expert consultation on human vitamin and mineral needs’, as opposed to 46.7% of participants in the control group [98].

An improved chance for acne was also observed to be due to the consumption of eggs [52], excess cola [89], soft drinks [99], corn [61], candy, fizzy drinks, fruit or fruit juices [65], cakes [100], and high-gluten diets [101].

## 4. Conclusions

The growing frequency of skin conditions, including acne vulgaris, presents challenges for not only community health but also for sufferers personally. Acne vulgaris is commonly the reason for a decreased quality of life, reduced appearance gratification, and feelings of well-being and personal assurance among sufferers. It can indicate a reduced social performance and depressed temperament; therefore, it is essential to know exactly what factors contribute to acne and to implement successful treatments to alleviate the symptoms of this dermatosis. The relationship of nutrition and acne is a topic that has been extensively discussed for many years. Whereas, in the history, eating behaviour has not been regarded as a demonstrated reason for acne in the medical literature, following an examination of the existing evidence, the researchers concluded that there is an increasing amount of scientific proof confirming the damaging effect of certain dietary components in the pathogenesis of acne lesions. Dairy products, chocolate, and saturated fats play a significant role in contributing to acne. Alcohol, salted products, gluten, eggs, biscuits, corn, fruit, sweets, cola, or soft drinks also appear to participate in the promotion and exacerbation of acne changes, but this needs to be confirmed by further research. There is a need for further research into the influence of diet on acne pathogenesis. However, these studies should be designed to avoid restrictions, including a low trial volume, absence of adequate controls, poor visual presentation, and inadequately reportable and inaccurate outcomes. Accurate knowledge of the products that negatively influence the development of this dermatosis will allow clinicians to provide their patients with appropriate recommendations and guidance to implement an elimination diet, ultimately reducing acne lesions and improving the quality of life for acne vulgaris sufferers.

## Figures and Tables

**Table 1:** Selected studies on the effect of milk and dairy products on the pathogenesis of acne vulgaris.

Research	Year	Methodology	Key Result	References
Adolescents				
Adebamowo C.A. et al.	2005	47,355 female participants responded and completed a questionnaire on frequency of eating in high school. This analysis includes women who fulfilled the questionnaire and replied to the question on doctor-diagnosed extreme teenage acne in 1989.	A positive association has been found between acne and the consumption of whole and skimmed milk. Cottage cheese and cream cheese are also positively related with acne.	[58]
Adebamowo C.A. et al.	2006	Using a questionnaire, 6094 girls of baseline age between 9 and 15 years from GUTS were surveyed.	Whole, low-fat, skimmed, or chocolate milk has been linked to acne.	[59]
Adebamowo C.A. et al.	2008	Using a questionnaire, 4273 boys of baseline age between 9 and 15 years from GUTS were surveyed.	A positive association between the consumption of skimmed milk and the occurrence of acne was proven. Full-fat milk, 2% milk, and low-fat milk were not associated with acne.	[60]
LaRosa C.L. et al.	2016	The study involved 225 participants aged 14 to 19 years with medium or no acne. Skin lesions were assessed and dietary history was taken using the Nutrition Data System for Research software.	Consumption of low-fat/low-fat milk, but not full-fat milk, was positively associated with acne incidence.	[63]
Okoro E. et al.	2016	A physical examination and analysis of data from interviews with 464 Nigerian students with an average age of 13.6 years was conducted.	The incidence of acne was higher in people who consumed milk like a beverage at least once a day.	[61]
Ulvestad M.	2017	A questionnaire-based study on baseline dairy intake and presence of acne after 3 years among 2489 Norwegian residents aged 15–16 years.	Consumption of at least two glasses of full-fat dairy products per day was associated with moderate to severe acne.	[55]
Alkhabbaz M. et al.	2020	A cross-sectional study was performed among 714 adolescents. The presence or absence of acne and acuteness was assessed using the Global Acne Grading Scale. Data on risk factors and potential confounders were collected from parents using a self-administered questionnaire and from adolescents by direct interview.	Factors that were not significantly associated with the development of acne vulgaris include semi-skimmed milk, full-fat cream cheese, low-fat cheese, white cheese, yoghurt, ice cream, and other dairy products.	[70]
Young adults (18–25 years old)				
Burris J. et al.	2014	The study enrolled 248 participants aged between 18 and 25 years (115 men and 133 women).	Compared to attendees with no acne or with mild acne, participants with medium to extreme acne reported intaking more portions of milk daily in their diet.	[65]
Pereira Duquia R. et al.	2017	The study assessed 2201 18-year-old men living in southern Brazil by a dermatologist.	The crude analysis showed a relationship between daily whole milk intake and the incidence of inflammatory acne, but this association was only found in the proximate analysis. Although the outcomes of the adjusted analyses were not statistically considerable, the prevalence rates of daily whole milk intake may indicate a poor positive relation with the incidence of inflammatory acne.	[64]
Pontes T. de C. et al.	2013	The target group consisted of gym-goers and patients of a dermatology clinic in João Pessoa (Brazil). The study included 30 participants aged 18–30 years. Acne severity was assessed before starting the supplements, and then one month and two months after starting the supplements.	This study showed the onset of acne with the gradual use of protein and calorie supplementation over a two-month period. This effect was more pronounced in women and in people without current acne and no family history of acne.	[46]
Adults				
Semedo D. et al.	2016	The study consisted of a questionnaire and physical examination in a sample of 1055 people aged between 20 and 60 who visited five healthcare centers in the Greater Porto area on random days.	Among the 1055 people surveyed, the prevalence of acne was estimated at 61.5%. Acne was associated with the intake of fully-fat milk and skimmed milk.	[56]
Juhl C.R. et al.	2018	We investigated the association between milk consumption, LCT-13910 C/T, and acne in 20,416 adults from the Danish General Population Survey (GESUS) using Mendel randomisation.	No observational or genetic link was found between milk consumption and acne	[69]
Penso L. et al.	2020	24,452 French adults completed an online questionnaire where they classified their acne condition. Associations between dietary behaviour and acne were investigated in multinomial logistic regression models. The survey was conducted as part of the NutriNet-Santé study.	A significant association was found between current acne and milk consumption.	[53]
Heng A.H.S. et al.	2022	3888 patients (2090 acne patients and 1798 controls) (17–71 years old) completed questionnaires and were examined by trained staff for severity of acne lesions and degree of scarring.	Frequent consumption of milk was associated with a protective effect for moderate acne, while frequent consumption of butter had a detrimental effect on the degree of acne scarring.	[68]
Different age range				
Di Landro A. et al.	2012	The study involved 568 participants (205 people with medium to extreme acne and 358 controls). The age of the subjects was between 10 and 57 years old. Acne escalation was determined using a global scale, and trained interviewers conducted interviews.	The risk of medium to severe acne rose with increased milk consumption in those consuming more than three portions per week. This connection was more pronounced for low-fat milk than whole milk.	[57]
Juhl C. R. et al.	2018	A metanalysis in which 14 studies were included, representing 78,529 people (23,046 patients with acne and 55,483 controls) aged between 7 and 30 years.	Any dairy such as milk, yoghurt, and cheese was associated with an increased odds quotient for acne.	[54]
Aalemi A. K. et al.	2019	Using the Global Acne Severity Scale, dermatologists assessed the acuteness of acne in 279 Kabul residents with acne and 279 control subjects. Participants were aged between 10 and 24 years. Eating habits were recorded using a questionnaire.	Intake of full-fat milk at least 3 days per week was related with moderate to severe acne incidence. The association with acne incidence was less pronounced for skimmed milk than for full-fat milk.	[52]
Kara Y. A. et al.	2020	Fifty-three acne patients and fifty-three controls took part in the study. The age of the subjects was between 13 and 44 years old. The subjects’ lesions were assessed and dietary composition was calculated.	In patients with acne vulgaris, it was observed that cheese consumption increased lesion formation.	[62]
Say Y.H. et al.	2021	1840 patients (1117 acne patients and 723 controls) completed questionnaires and were examined by trained staff for severity of acne lesions and degree of scarring.	Frequent consumption (most or all days) of products commonly eaten at breakfast (butter, milk, probiotic cartridges, and cereals) reduced the risk of acne and larger acne scars.	[67]

**Table 2:** Selected studies on the effect of chocolate on the pathogenesis of acne vulgaris.

Research	Year	Methodology	Key Result	References
Caperton et al.	2014	14 men aged 18 to 35 years with a history of acne vulgaris were assigned to swallow capsules filled with unsweetened 100% cocoa, hydrolysed gelatine powder, or a combination of both. Acne changes were assessed at the start of the study, on day four, and on day seven of the study.	There was a positive Pearson correlation coefficient of low strength between the amount of chocolate consumed by each participant and the number of lesions developed in each patient between baseline and day 4, while there was a positive correlation of medium strength between baseline and day 7.	[81]
Vongraviopap S. et al.	2015	25 acne-prone men consumed 25 g of dark chocolate (99% cocoa) daily for four weeks. The severity of acne lesions at the start of the study, and after two and four weeks of the study were assessed.	Statistically significant changes in acne scores and the number of blackheads and inflammatory papules were detected after 2 weeks. After 4 weeks, changes remained statistically significant compared to baseline values.	[84]
Chalyk N. et al.	2018	33 men (17 young men and 16 middle-aged men) consumed 10 g per day of dark chocolate (70% cocoa) for 4 weeks. Morphological characteristics of residual skin surface components (RSSCs) were assessed before the introduction of chocolate into the diet and after consumption for 4 weeks. Samples were assessed by staining with haematoxylin and eosin, Oil Red O, and crystal violet solution (Gram staining).	Chocolate consumption increased corneocyte exfoliation in both age groups, but the increase was statistically significant in the young male group. A significant increase in the presence of Gram-positive micro-organisms on the skin surface was observed.	[72]
Suppiah T. et al.	2018	The severity of acne lesions was assessed and a questionnaire interview on eating habits was conducted among 57 subjects with acne vulgaris and 57 controls. All subjects were 14 years of age or older.	Chocolate consumption was significantly higher among sick people with acne vulgaris compared to controls.	[82]
Karadağ A. et al.	2019	The study involved 3826 patients with acne vulgaris and 759 control patients. A dermatologist assessed the severity of the skin lesions and a questionnaire evaluating the subjects’ dietary habits was administered.	A positive correlation was found between chocolate consumption and the severity of acne lesions.	[83]

**Table 3:** Selected studies on the effect of fatty foods on the pathogenesis of acne vulgaris.

Research	Year	Methodology	Key Result	References
Wei B. et al.	2010	Using a questionnaire, data were collected from 5696 adolescents in north-eastern China (2920 patients and 2776 controls).	Acne risk factors were identified, among which fried foods were listed.	[88]
Abo El-Fetoh N. M. et al.	2016	Four hundred male students from Arar (Kingdom of Saudi Arabia) were recruited for the research. Figures were collected by personal discussion and survey methods.	Consumption of fatty foods as a risk factor for acne vulgaris.	[89]
Aalemi A. K. et al.	2019	Using the Global Acne Severity Scale, dermatologists assessed the severity of acne in 279 Kabul residents with acne and 279 control subjects. Participants were aged between 10 and 24 years. Eating habits have been recorded with the help of a survey.	Intake of products such as crisps, pizza, or red meat had a positive correlation with the occurrence of acne lesions.	[9]
Penso L. et al.	2020	24,452 French residents completed an online questionnaire where they classified their acne condition. Links of dietary habits and acne were investigated in multinomial logistic regression models. The survey was conducted as element of the NutriNet-Santé study.	A significant association was found between current acne and the consumption of oily products.	[54]
